# How low working memory demands and reduced anticipatory attentional gating contribute to impaired inhibition during acute alcohol intoxication

**DOI:** 10.1038/s41598-022-06517-9

**Published:** 2022-02-21

**Authors:** Ann-Kathrin Stock, Shijing Yu, Filippo Ghin, Christian Beste

**Affiliations:** 1 Cognitive Neurophysiology, Department of Child and Adolescent Psychiatry, Faculty of Medicine, TU Dresden, Schubertstrasse 42, 01309 Dresden, Germany; 2 University Neuropsychology Center, Faculty of Medicine, TU Dresden, Dresden, Germany; 3 Biopsychology, Faculty of Psychology, TU Dresden, Dresden, Germany

**Keywords:** Cognitive neuroscience, Human behaviour

## Abstract

High-dose alcohol intoxication is commonly associated with impaired inhibition, but the boundary conditions, as well as associated neurocognitive/neuroanatomical changes have remained rather unclear. This study was motivated by the counterintuitive finding that high-dose alcohol intoxication compromises response inhibition performance when working memory demands were low, but not when they were high. To investigate whether this is more likely to be caused by deficits in cognitive control processes or in attentional processes, we examined event-related (de)synchronization processes in theta and alpha-band activity and performed beamforming analyses on the EEG data of previously published behavioral findings. This yielded two possible explanations: There may be a selective decrease of working memory engagement in case of relatively low demand, which boosts response automatization, ultimately putting more strain on the remaining inhibitory resources. Alternatively, there may be a decrease in proactive preparatory and anticipatory attentional gating processes in case of relatively low demand, hindering attentional sampling of upcoming stimuli. Crucially, both of these interrelated mechanisms reflect differential alcohol effects after the actual motor inhibition process and therefore tend to be processes that serve to anticipate future response inhibition affordances. This provides new insights into how high-dose alcohol intoxication can impair inhibitory control.

## Introduction

Drug consumption is a significant problem in most societies. Aside from tobacco, alcohol is one of the most frequently used drugs^1^. Excessive alcohol use can lead to the development of alcohol use disorder (AUD), and many research efforts are undertaken to identify (neural) mechanisms that could be targeted in order to regain control over drug intake in AUD^2^. However, research aiming to understand how cognitive functions are affected by acute alcohol intoxication or binge drinking is necessary to better understand how dysfunctions arise and to develop strategies for regaining control over drug intake. This becomes all the more relevant as frequent binge drinking seems to increase the likelihood of developing AUD^3,4^.

One of the critical cognitive functions associated with the (loss of) control over alcohol consumption is ‘inhibitory control’^5^. There are many facets of inhibitory control^6^, but one major relevant facet in alcohol and addiction research is the inhibition of prepotent responses^7,8^. Put simply, response inhibition is easy to accomplish whenever cognitive control resources are high and/or there is only a weak tendency towards executing an unwanted response. Conversely, inhibition failures become more likely when cognitive control resources are low and/or there is a strong tendency towards a wrong response^9–12^. In the past years, several studies focused on how high-dose alcohol intoxication (i.e., of ~ 1.2‰, or 120 mg/dl) affects response inhibition^13–16^. At this stage, drinkers typically tend to experience anxiolytic effects and behavioral disinhibition, but only mild sedation. Cognition and judgement may be impaired, but most drinkers do not (yet) experience marked ataxia or motor impairments^17^. Previous intoxication studies^13–16^ have shown that at ~ 1.2‰ (120 mg/dl), participants are still able to comply with task instructions and rarely suffer from adverse side effects such as nausea or vomiting. While most studies showed that high-dose alcohol intoxication led to ubiquitous impairments in response inhibition, some counterintuitive findings challenged this picture. For example, Stock et al.^16^ showed that high-dose intoxication effects on response inhibition depend on mental workload. This mental workload was operationalized via the different number of target stimulus features required to differentiate between Go and Nogo responses in two different blocks as well as by the spatial rotation of target stimuli in both task blocks. They showed that compared to a sober condition, alcohol intoxication compromised inhibitory control in conditions with low working memory demand. However, this negative effect of alcohol compared to a sober state entirely vanished when working memory load was relatively high during response inhibition^16^. The finding that the difference in alcohol effects (i.e., the contrast between the sober and intoxicated state) depends on working memory load should be examined in more detail to better understand the boundary conditions of high-dose alcohol intoxication effects. This especially applies to the underlying neurophysiology and functional neuroanatomy, as that information is key to developing and optimizing non-invasive brain stimulation approaches, which are increasingly considered in the treatment of addictions^18,19^.

Against this background, the current study analyzed the neurophysiology of working memory load-dependent differences in alcohol effects during response inhibition using EEG-beamforming. Employing EEG-beamforming, it is possible to delineate the functional neuroanatomy associated with modulatory effects in specific frequency bands^20^. We focused on EEG theta and alpha frequency bands. Theta oscillations are well-known for their crucial role in cognitive control functions, including inhibition and working memory operations^21–23^. Alpha oscillations, and alpha desynchronization processes, in particular, have also been associated with working memory processes. Furthermore, they have been associated with a gating mechanism that controls access to (working) memory^23–25^ to enable working memory maintenance and protection against interfering information^26^. Given these functional roles of theta and alpha band activity, we hypothesized that the response inhibition difference in alcohol effects under low vs. high working memory demands should be reflected by these frequency bands. We calculated event-related synchronization and desynchronization processes^27^ since the functional role of alpha-band activity is mainly conceptualized in terms of relative synchronization and desynchronization processes^23–25^. Event-related synchronization (as opposed to desynchronization) in the EEG is an index of reduced activity in the examined frequency band^27^. Likely, working memory-associated differences in alcohol effects on inhibition are expressed in a relative synchronization of alpha and theta frequency band dynamics. These effects are likely to occur during the actual process of response inhibition. Several lines of evidence show that EEG correlates of the actual process of inhibitory control, including those using the same experimental paradigm^28,29^, occur in the time interval of up to 600 ms after the presentation of the stimulus intended to trigger inhibitory control^30^. Inhibitory control is mediated via a distinct cortical network encompassing the superior and middle frontal gyrus and the inferior frontal gyrus^6^. Since prefrontal structures are also central for working memory^31–34^, prefrontal structures likely reflect differences in alcohol effects on response inhibition under low vs. high working memory demands.

However, it cannot be excluded that relevant modulations of neurophysiological processes occur in entirely different time windows and not during the actual process of motor inhibition. The reason behind this is that behavioral deficits related to alcohol effects might also arise from changes in attentional focus and processing (often termed “alcohol myopia”)^35–37^. Attentional anticipatory processes play a central role in proactive control processes that help to prepare the cognitive system for upcoming demands^38^. Particularly, desynchronized alpha-band activity is essential for anticipatory attentional gating and the updating of relevant upcoming information^39,40^. Due to the sequential structure of experimental paradigms used to examine response inhibition processes in this study, such anticipatory attentional gating mechanisms are likely to play an important role. Intriguingly, recent data on response inhibition have shown that anticipatory proactive control processes occurring in-between a completed response inhibition, and before an upcoming occasion to re-engage in response inhibition, have a substantial impact on inhibitory performance^41^. Therefore, it is also possible that differences in alcohol effects between response inhibition under low and high working memory demands are reflected by theta and alpha synchronization processes after the actual inhibitory control process has been finished. If this is the case, it likely that instead of prefrontal structures, cortical areas associated with bottom-up attentional processing, such as occipital structures, are most associated with modulations in theta and alpha band synchronization processes.

## Results

### Sample description and intoxication results

The participants were on average 24.2 ± 0.7 years old, 182.7 ± 1.2 cm tall, and weighed 78.2 ± 2.5 kg. Based on this, they received on average 92.0 ± 1.8 g of alcohol, equaling 287.4 ± 5.6 ml of vodka (40% alcohol by volume). The consumption resulted in a mean BrAC of 1.01 ± 0.06‰ (101 mg/dl) when starting the experiment and 1.08 ± 0.04‰ (108 mg/dl) when finishing the experiment.

### Behavioral results

**Figure 1:**
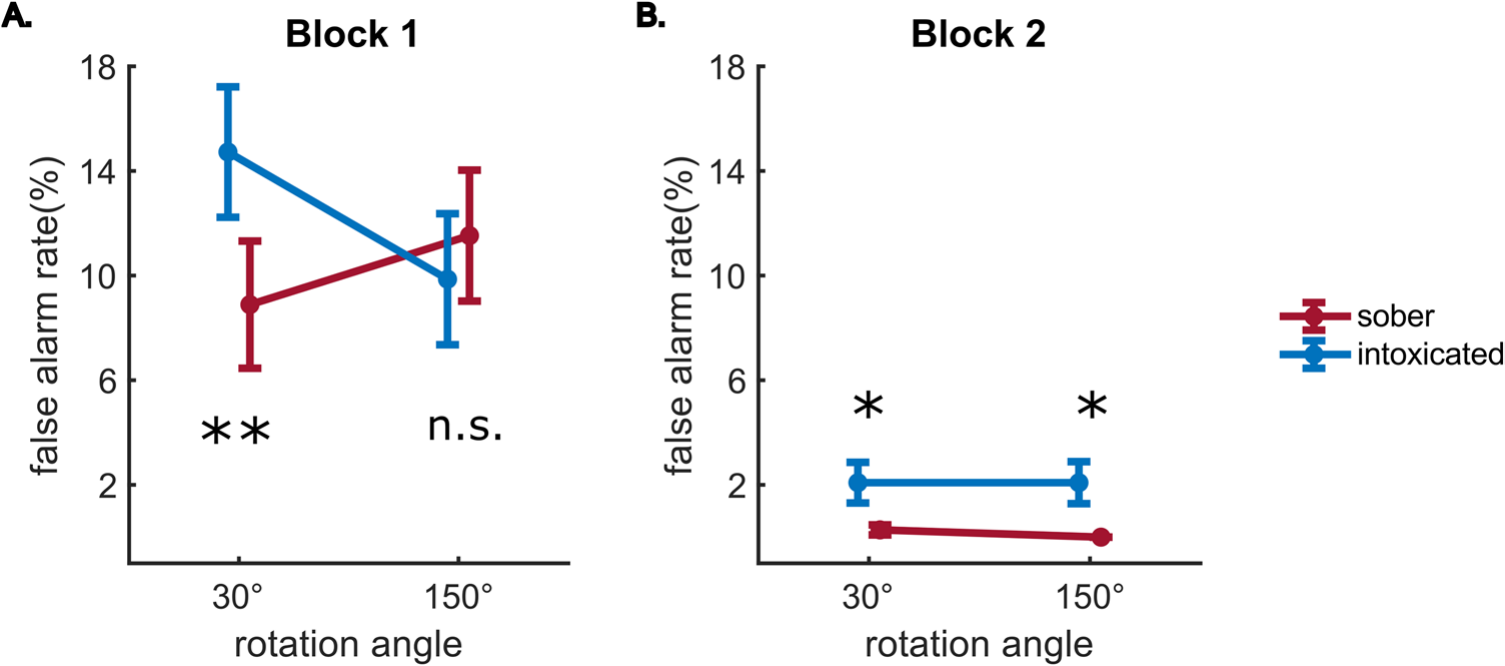
Behavioral results of Nogo trials. (**A,B**) Present the respective false alarm rates in block 1 and block 2. Error bars indicate the standard error of the mean. ‘*’ and ‘**’ indicate the significance of intoxication effects (sober vs. intoxicated) at p ≤ 0.05 and p ≤ 0.01, respectively. ‘n.s.’ indicates no significant intoxication effect.

Behavioral results for the Nogo trials are presented in Fig. 1.

The repeated measures ANOVA revealed a main effect of “block” (F(1,19) = 27.40, p < 0.001, ${\eta }_{p}^{2}$ = 0.59), with higher false alarm rates in the most demanding block 1 (11.25% ± 2.01) than in the least demanding block 2 (1.11% ± 0.36). Since not all false alarm rates were normally distributed, we confirmed this difference with a Wilcoxon test for paired samples (p < 0.001). An interaction effect of “intoxication × rotation angle” was observed (F(1,19) = 7.37, p = 0.014, ${\eta }_{p}^{2}$ = 0.28), and the interaction of “intoxication × rotation angle × block” was also significant (F(1,19) = 7.46, p = 0.013, ${\eta }_{p}^{2}$ = 0.28). This latter interaction was analyzed using post-hoc tests. The post-hoc tests were conducted separately for each block by applying repeated measures ANOVAs using the within-subject factors “intoxication” and “rotation angle”. In block 2, only “intoxication” revealed a main effect (F(1,19) = 7.39, p = 0.014, ${\eta }_{p}^{2}$ = 0.28), showing higher false alarm rates in the intoxicated condition (2.09% ± 0.71), compared to the sober condition (0.14% ± 0.10). This was confirmed by an additional Wilcoxon test (p = 0.003). In block 1, only the interaction of “intoxication × rotation angle” was significant (F(1,19) = 7.91, p = 0.011, ${\eta }_{p}^{2}$ = 0.29). Separate Wilcoxon tests comparing the sober and intoxicated conditions in block 1 showed that the false alarm rate was significantly lower in the sober than in the intoxicated status for the 30° rotation condition (p = 0.006, sober = 8.89% ± 2.43, intoxicated = 14.72% ± 2.49), but not for the 150° condition (p = 0.413).

The behavioral raw data and analyses are available on the open science framework (https://osf.io/wbafc/?view_only=e98d0f3c75e14c8992ec24c4e3bb8b7f).

### Neurophysiological results: theta band activity

**Figure 2:**
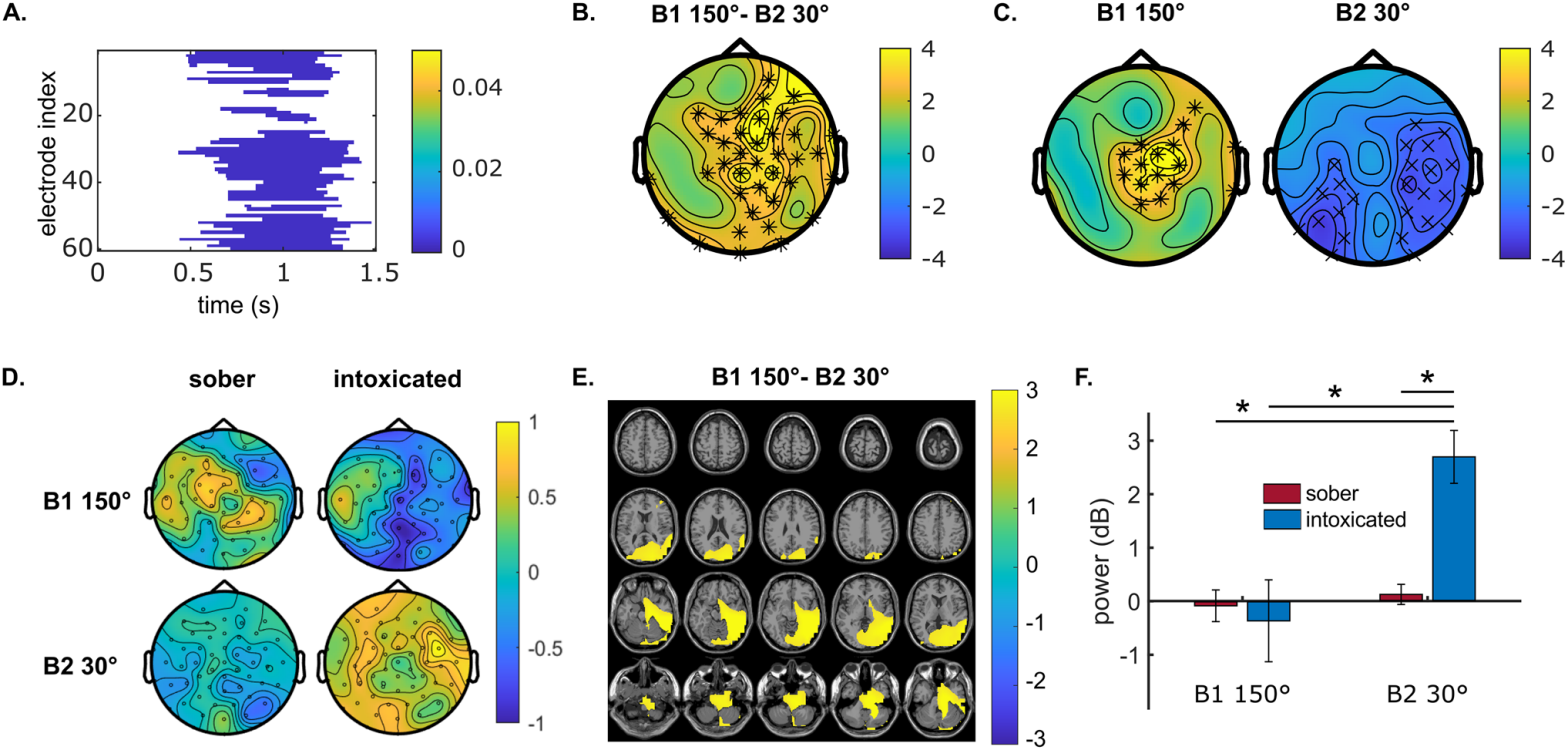
Neurophysiological results for task-related theta activity in Nogo trials. (**A**) Shows the cluster-based permutation test result of comparing intoxication effects (sober-intoxicated) between the conditions of block 1 at 150° and block 2 at 30° in each electrode and time point for 0–1.5 s after stimulus presentation. Only significant results (p ≤ 0.5) are presented in color. The color bar indicates p-values. (**B**) Shows the topographical map of the intoxication effect difference (in dB) between the conditions of block 1 at 150° and block 2 at 30° in the time window of 0.75–1.5 s. (**C**) Presents the topographical maps of intoxication effects in conditions of block 1 at 150° and block 2 at 30° separately. In plots (**B,C**), ‘×’ and ‘*’ label the electrodes with significant differences at p ≤ 0.05 and p ≤ 0.01, respectively. Color bars indicate the t-values of cluster-based permutation tests. For plot (**B**), warm color suggests a higher intoxication effect in the block 2 at 30° than in the block 1 at 150° condition, and the cold color suggests the opposite. For plot (**C**), warm and cold colors indicate the positive and negative contrasts (sober-intoxicated) of task-related theta band power. (**D**) Shows the topographical maps of task-related theta activities in the original conditions. The color bar indicates baseline-normalized power in dB. (**E**) Shows the anatomical regions where strong differences in the intoxication effect were observed between the block 1 at 150° and the block 2 at 30°. The color bar indicates the task-related theta band power difference in dB. The warm color suggests a stronger intoxication effect in the block 2 at 30° condition than in the block 1 at 150° condition. (**F**) Represents the averaged source-level theta powers in dB in each condition for the regions depicted in (**E**). Each error bar indicates the standard error of the mean. All the results in plots (**B–F**) were derived from data between 0.75 and 1.5 s after stimulus presentation.

The neurophysiological results for task-related theta band activity in Nogo trials are shown in Fig. 2.

At the electrode level, the cluster-based permutation test (see step 3 in Fig. 5) revealed significant differences in intoxication effects (sober-intoxicated) between conditions B2 30° and B1 150° in most electrodes for task-related theta activity. These differences were mainly observed from 0.5 to 1.5 s after stimulus onset (see Fig. 2A). However, the time window showing the most activity differences between the theta and the alpha band was between 0.75 and 1.5 s after stimulus presentation. Therefore, further data analyses focused on this time window. Figure 2B shows that the intoxication effect in the cognitively least demanding condition (B2 30°) was significantly stronger than in the most demanding condition (B1 150°), and the significant differences were obtained at central and posterior electrode sites (results from step 5 in Fig. 5). Figure 2C presents the individual intoxication effects in the above conditions (see step 7 in Fig. 5). In the B2 30° condition, the task-related theta band activity was more desynchronized in the sober status than in the intoxicated status, especially at temporal and posterior electrode sites. In contrast, for the B1 150° condition, theta band activity was more synchronized in the sober status than in the intoxicated status at central electrodes. We then averaged the time–frequency powers from 0.75 to 1.5 s across time (see step 6 in Fig. 5), which returned the theta band synchronization/desynchronization in the selected time window for the four original conditions. This showed that task-related theta frequency was barely evident in the sober B2 30° condition. In contrast, theta synchronization was strong in the intoxicated B2 30° condition. In the sober B1 150° condition, task-related theta activity was synchronized at left fronto-central and right postero-central electrodes. In the intoxicated B1 150° condition, it was mainly desynchronized except for left temporal electrode sites.

For the source level (Fig. 2E), the difference in theta band intoxication effects between conditions B1 150° and B2 30° was mainly observed in the right middle, inferior and superior temporal gyrus (BA 21, 20, 41, 42), right fusiform gyrus (BA 37) and in the middle, superior and inferior occipital gyrus (BA 18, 19, 17). Moreover, areas between the temporal and occipital cortex, such as the lingual gyrus, calcarine fissure, and cuneus (BA 17), differed. This difference also expanded to the right hippocampus and parahippocampal gyrus (BA 28, 27). As illustrated in Fig. 2F, Wilcoxon tests for paired samples comparing all conditions revealed significantly stronger source-level theta synchronization in the intoxicated B2 30° condition than in the other three conditions (all p ≤ 0.001). However, no significant difference was detected among the three other conditions (all p ≥ 0.526).

### Neurophysiological results: alpha-band activity

**Figure 3:**
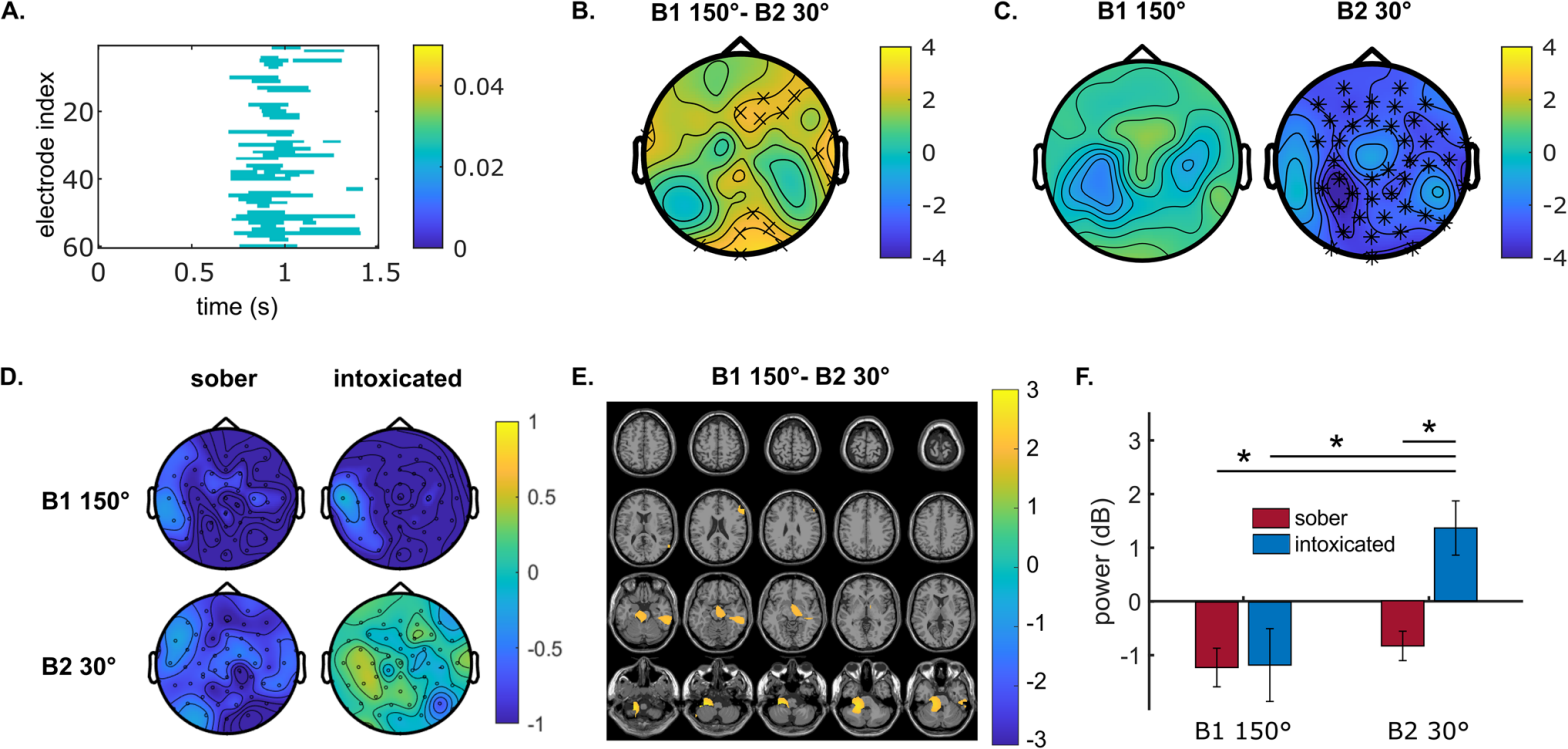
Neurophysiological results for task-related alpha activity in Nogo trials. (**A**) Shows the cluster-based permutation test result of comparing intoxication effects (sober-intoxicated) between the conditions of block 1 at 150° and block 2 at 30° in each electrode and time point for 0–1.5 s after stimulus presentation. Only significant results (p ≤ 0.5) are presented in color. The color bar indicates p-values. (**B**) Shows the topographical map of the intoxication effect difference between the conditions of block 1 at 150° and block 2 at 30° in the time window of 0.75–1.5 s. (**C**) Presents the topographical maps of intoxication effects in the conditions of block 1 at 150° and block 2 at 30° separately. In plots (**B,C**), ‘×’ and ‘*’ label the electrodes with significant differences at p ≤ 0.05 and p ≤ 0.01, respectively. Color bars indicate the t-values of cluster-based permutation tests. For plot (**B**), warm color suggests a higher intoxication effect in the block 2 at 30° than in the block 1 at 150° condition, and the cold color suggests the opposite. For plot (**C**), warm and cold colors indicate the positive and negative contrasts (sober-intoxicated) of task-related alpha band power. (**D**) Shows the topographical maps of task-related alpha activities in the original conditions. The color bar indicates baseline-normalized power in dB. (**E**) Shows the anatomical regions where strong differences in the intoxication effect were observed between the conditions of block 1 at 150° and block 2 at 30°. The color bar indicates the task-related alpha band power difference in dB. The warm color suggests a stronger intoxication effect in the block 2 at 30° condition than in the block 1 at 150° condition. (**F**) Represents the averaged source-level alpha powers in dB in each condition for the regions depicted in (**E**). Each error bar indicates the standard error of the mean. All the results in plots (**B–F**) were derived from data between 0.75 and 1.5 s after stimulus presentation.

The neurophysiological results for task-related alpha activity in Nogo trials are presented in the same way as for theta activity (see Fig. 3). Significant differences in intoxication effects between conditions B1 150° and B2 30° (Fig. 3A) were detected at the electrode level around 0.75 to 1.5 s after stimulus presentation (see step 3 in Fig. 5). The cluster-based permutation test revealed a larger intoxication effect in the B2 30° condition, as compared to the B1 150° condition, but only in a few electrodes at right frontal and posterior sites (Fig. 3B). Figure 3C shows the results of comparing the sober and the intoxicated status in the B2 30° and B1 150° conditions separately (see step 7 in Fig. 5). The intoxication effect was evident in the B2 30° condition with more task-related alpha synchronization in the intoxicated state than in the sober state. However, the intoxication effect in the B1 150° condition was not significant. Figure 3D shows the average alpha synchronization/desynchronization across the selected time window for the original four conditions using time–frequency powers (see step 6 in Fig. 5). In the sober B2 30°, sober B1 150°, and intoxicated B1 150° conditions, task-related alpha desynchronization was evident at almost all electrodes. In the intoxicated B2 30° condition, however, a task-related alpha synchronization was evident.

**Figure 4:**
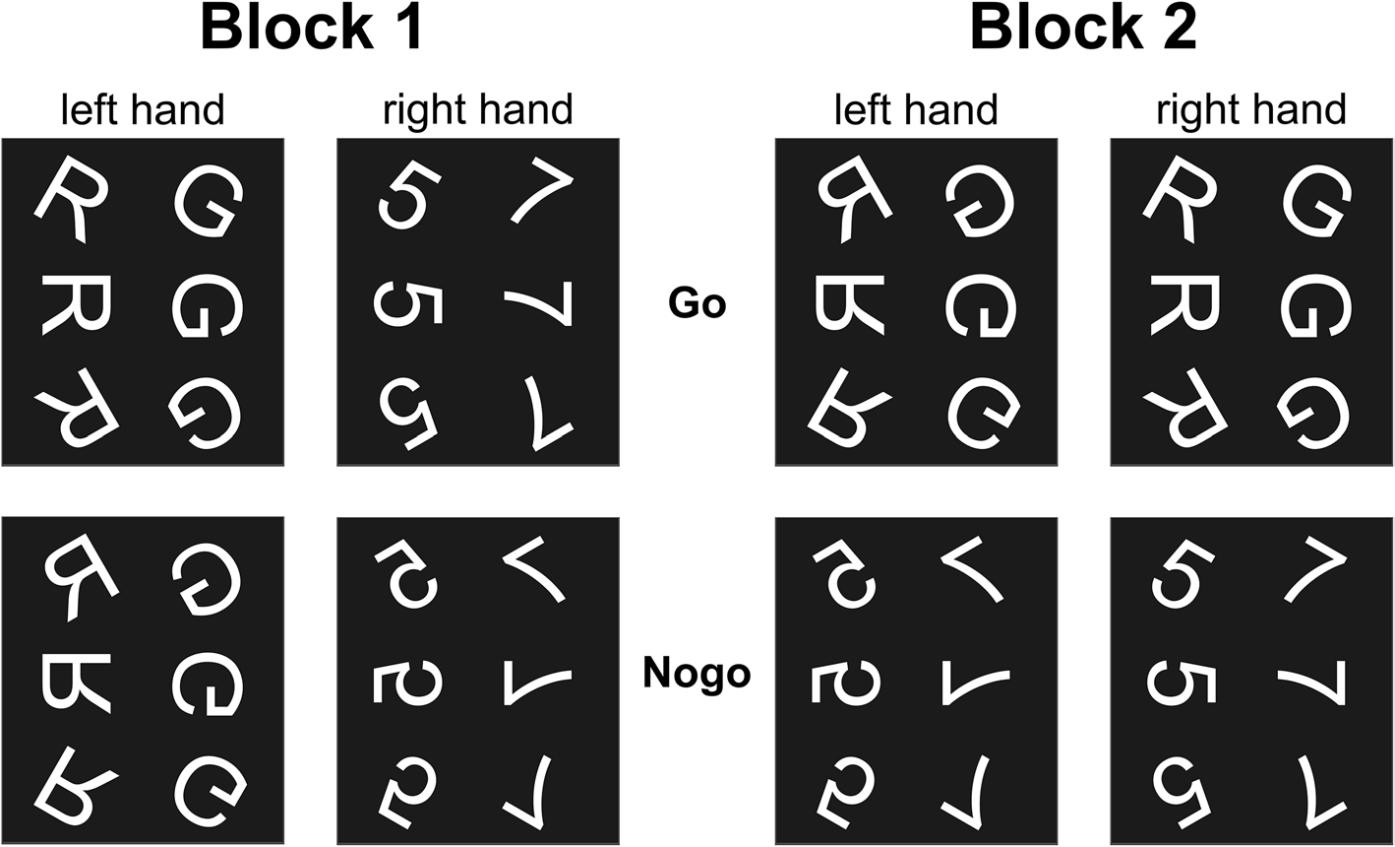
Experimental paradigm. Each panel represents one type of stimuli. The required responses to Go stimuli are provided in the top row. Nogo trials, which did not require a motor response, are illustrated in the bottom row.

The source level task-related alpha-band activity (Fig. 3E) revealed that the intoxication effect difference between conditions B1 150° and B2 30° was mainly right-lateralized to the inferior temporal gyrus (BA 20), the fusiform gyrus (BA 37), and the triangular part of inferior frontal gyrus (BA 45). In the parahippocampal gyrus and the right hippocampus (BA 27, 28), the sober-intoxicated contrast was larger in the B1 150° than in the B2 30° condition. In the Wilcoxon tests for paired samples comparing all conditions, source-level alpha synchronization was significantly stronger in the intoxication B2 30° condition compared to all other conditions (all p ≤ 0.003), as illustrated in Fig. 3F. The comparisons between the other three conditions did not show any significant differences (all p ≥  0.526).

## Discussion

The current study examined neurophysiological processes underlying differential high-dose alcohol intoxication effects on inhibitory control depending on working memory demands. This study was motivated by previous behavioral findings^16^, which provided the counterintuitive finding that high-dose alcohol intoxication compromised response inhibition performance when demands on working memory processes were low, but not when they were highly taxed. On the behavioral level, this finding can be explained by the fact that high demands on response selection hinder response automatization, thus ultimately lowering response inhibition demands^16^. Against the background of different theories, we aimed to examine whether this variation of behavioral intoxication effects is more likely to be caused by deficits in cognitive control processes or in attentional processes. To determine which neurophysiological processes and functional neuroanatomical structures are associated with these counterintuitive effects, we examined event-related synchronization and desynchronization processes^27^ in theta and alpha-band activity and performed beamforming analyses. Of note, the investigated sample largely overlaps with previously published behavioral data^16^, while the EEG data have not been previously published. Specifically, we used the data of all participants included in the previous study as well as the data of two additional participants who had previously been excluded due to lower Go response accuracy (which was not analyzed and therefore not considered in the current study).

Despite those slight differences between the composition of the investigated samples and despite the fact that we only analyzed pre-intoxication data to assess sober performance, the behavioral data pattern does not differ from the previously reported behavioral findings^16^. The detrimental effect of binge drinking was significant in the easy conditions characterized by a relatively low working memory demand (B2 30°, B2 150°, and B 30°). However, it vanished in the most challenging condition, characterized by a relatively high working memory demand (B1 150°). Alcohol-induced declines in inhibitory control have frequently been reported^42–44^, but this seemingly contradictory finding provided new and exciting insights into how and when alcohol impairs response inhibition: Successful inhibition does not only depend on the available cognitive control capacities^45,46^, but also on how prepotent the tendency towards an incorrect or unwanted response is^9–12,47^. Put simply, inhibition is easy to accomplish whenever cognitive control resources are high and/or there is only a weak tendency towards executing an unwanted response. Conversely, inhibition failures become more likely when cognitive control resources are low and/or there is a strong tendency towards a wrong response. In the context of the investigated task, working memory load did not only increase task difficulty, but it also ultimately delayed Go response speed and automatization. Against this background, high working memory load / task difficulty hinder response automatization to such a degree that the residual inhibitory capacities retained during alcohol intoxication are still sufficient to enable response inhibition at the same level as sober. In contrast, seemingly effortless and non-demanding tasks with less working memory load result in comparatively fast and strong Go response automatization, which can no longer be matched by the participants’ weakened cognitive control during intoxication. As a result, intoxicated individuals show significantly more inhibition failures in the seemingly straightforward task but not in the cognitively most challenging task condition^16^. While this explanation helps to understand the effects from a theoretical point of view, it has however remained unclear which sub-process and brain regions give rise to the differential alcohol-induced inhibition impairments. Given the repeated reports of cognitive control (especially inhibition) failures during alcohol intoxication^13,15,48^, it is commonly assumed that alcohol has a detrimental effect on cognitive control functions. There is, however, a competing approach which assumes that those behavioral deficits might arise from changes in attentional focus and processing (often termed “alcohol myopia”)^35–37^.

With respect to this question, neurophysiological data can be used to refine the initial interpretation and provide more detailed answers to how and when alcohol-induced inhibition effects emerge. For the neurophysiological data analysis, we focused on the difference between alcohol effects found in the task conditions with the lowest working memory load, which should result in the highest degree of response automatization (B2 30°), and the highest working memory load, which should result in the lowest degree of response automatization (B1 150°), because this contrast maximizes neurophysiological effects and provides the most reliable insights into neurophysiological dynamics. We did not analyze the mere alcohol effect (i.e., differences between the sober and intoxicated state) and instead focussed on condition-associated differences in the alcohol effect itself to get to the bottom of the differential behavioral effect. Therefore, the reported results do not reflect when and where alcohol had the biggest effect on the neurophysiological signals, but instead when and where the alcohol effect differed most between the affected and the unaffected task condition.

The most striking finding of these analyses is that differential alcohol effects between response inhibition trials with low vs. high working memory demand occur in theta and alpha processes in a time interval between 500 and 1500 ms after stimulus presentation. Numerous findings on EEG correlates of inhibitory control, including those using the same experimental paradigm^28,29^, suggest that processes underlying motor inhibition occur in the time interval of up to 600 ms^30^. This suggests that it is not the modulation of sub-processes directly involved in motor inhibition, which drives the observed differential effects. Instead, processes after the actual trial performance seem to best reflect the differential effects of high-dose alcohol intoxication on trials with different response inhibition requirements. While this may seem a little counterintuitive at first, it is essential to note that working memory demands and thus automatization tendencies were not only modulated by the different stimulus rotations angles, but also by the two different task blocks, as the distinction between Go and Nogo was much easier in block 2 than in block 1 (compare methods section/Fig. 4). Hence, any differences observed after task performance do not only reflect differences in the previous trial, but also differences in how the next trial may be approached in block 1 vs. block 2. Overall, our findings point to a new mechanism by which high-dose alcohol intoxication can impair inhibitory control: As shown by positive dB-normalized power values^27,49^, alcohol intoxication was associated with more synchronized (and thus less effective) neural activity in both the theta and the alpha frequency bands, but only in the B2 30° condition, which was characterized by significantly worse behavioral inhibition during acute intoxication. In contrast, no such effect was evident in the B1 150° condition, which was characterized by the lack of detrimental behavioral effects during acute intoxication. Theta oscillations are well-known for their crucial role in cognitive control functions, including inhibition and working memory operations^21–23,50^. Alpha oscillations have been associated with working memory processes as well, but also with a gating mechanism controlling access to (working) memory^23–25^ to enable working memory maintenance and protection against interfering information^26^. For both theta and alpha band synchronization processes, regions in the inferior temporal gyrus and the parahippocampal gyrus were associated with the differential effects of alcohol intoxication across the investigated task conditions. These functional neuroanatomical regions are well-known to serve working memory processes^51,52^, matching the assumption that the differential alcohol effects across conditions were rooted in working memory-related differences. As event-related synchronization in the EEG is an index of reduced activity in the examined frequency bands^27^, these results suggest that whenever working memory demands are low during inhibitory control, high-dose alcohol intoxication reduces theta and alpha activity in neuroanatomical structures relevant for those working memory processes. Possibly, high doses of alcohol push working-memory relevant functional neuroanatomical structures into a state of relative inactivity whenever this function is not highly taxed. Due to this alcohol-induced inactivity in working-memory-relevant structures, theta and alpha frequency-dependent working memory processes cannot easily be re-activated in the upcoming trial, and sequential (trial-based) encoding of information relevant for inhibitory control is hampered. This will likely lead to a further reduction in the invested working memory resources, possibly also due to an increased Go response automatization tendency, which further increases the demand on cognitive control functions. Therefore, task performance declines.

However, another not mutually exclusive interpretation is also possible. Aside from structures closely related to working memory processes, the occipital and right inferior frontal cortices also revealed differences. As the stimuli were presented for about 1100 ms (or until button press) before the fixation period of the subsequent trials began, differences in theta and alpha band activity are evident in a time interval in which preparatory processes for the upcoming trial are likely to occur. Since the upcoming trial likely imposes demands on cognitive control (including response inhibition processes), it is possible that proactive control processes—referring to a sustained and anticipatory goal-driven state^38^ come into play. Desynchronized alpha-band activity is essential for anticipatory attentional gating and updating mechanism of relevant upcoming information^39,40^. However, the current data show more synchronized alpha-band activity in the impaired A30° condition, suggesting that anticipatory attentional gating and updating of relevant upcoming information was likely dysfunctional when working memory demands are low, leading to greater response automatization. Notably, the beamforming analyses revealed differences in inferior frontal cortices associated with alpha-band synchronizations. This region is not only involved in inhibitory control^53^ but has also been associated with target detection and attentional sampling^54,55^. Similarly, theta band activity in sensory regions like the observed occipital regions serves attentional functions. Several lines of evidence suggest information sampling follows a theta-rhythm^56,57^ and theta band activity has also been related to pro-active preparatory processes^41^. The observed event-related synchronization (deactivation) of theta and alpha-band activity in occipital and inferior frontal regions may thus reflect differential impairments in attentional sampling and anticipatory attentional gating processes. The alcohol-induced relative inactivity of the theta and alpha frequencies in the period between 750 and 1500 ms after stimulus presentation may thus hinder attentional sampling of relevant upcoming stimuli when task demands are low and participants have a strong tendency towards response automatization (i.e., in block 2). Therefore, task performance declines.

With respect to potential limitations, it should be noted that we did not obtain ethical permission to include female participants, so that the question of sex differences cannot be answered. Secondly, the order of both the task blocks and appointments was always the same. Yet, additional analyses provided in the Supplementary Material did not evidence any effects of stimulus reassignment from block 1 to block 2. As for the appointment order, none of the assessed performance measures seems to have significantly improved on the second (intoxicated) appointment. Therefore, it seems unlikely that the observed intoxication effects were mainly driven by learning effects. However, we cannot exclude the possibility that intoxication effects might become larger if the appointment order was reversed.

Taken together, differential effects of alcohol intoxication on response inhibition in case of low-vs. high working memory demands may emerge due to two possibly connected neural mechanisms reflected by theta and alpha synchronization processes. One mechanism may be a selective decrease of working memory engagement in case of relatively low demand, which may further boost response automatization and thus put more strain on the remaining inhibitory resources. The other mechanism may be a decrease in proactive preparatory and anticipatory attentional gating processes in case of relatively low demand, which may hinder attentional sampling of upcoming stimuli. Crucially, both of these interrelated mechanisms best reflect the differential alcohol effects after the actual motor inhibition process and therefore tend to be processes that serve to anticipate future response inhibition affordances. The findings point to a novel mechanistic facet by which high-dose alcohol intoxication can impair (cognitive) inhibitory control.

## Methods

### Experimental subjects and ethical approval

N = 20 healthy male volunteers (19 to 32 years old, all right-handed) participated in the current study. The data was taken from the sample of a previous study on the same task, which provided behavioral results, but did not investigate the EEG signal, because the methods employed in the current study were not available to us at that time^16^. Specifically, we used the data of all participants included in the previous study plus two additional participants who had previously been excluded due to lower Go response accuracy (yet still over > 60%). As Go responses were not analyzed in the current study, we decided to not exclude those participants from our current analyses. All participants had normal or corrected-to-normal vision and reported no illnesses or current intake of relevant medication. Each participant reached a score between 1 and 15 in the alcohol use disorder identification test (AUDIT)^58^, indicating low to moderate risk of alcohol addiction and low probability of alcohol tolerance. All participants provided written informed consent before the experiment and received reimbursements (10€ per full hour) after the experiment. The ethics committee of the Medical Faculty of the TU Dresden approved the study (EK 293082014), and all procedures followed the Declaration of Helsinki.

**Figure 5:**
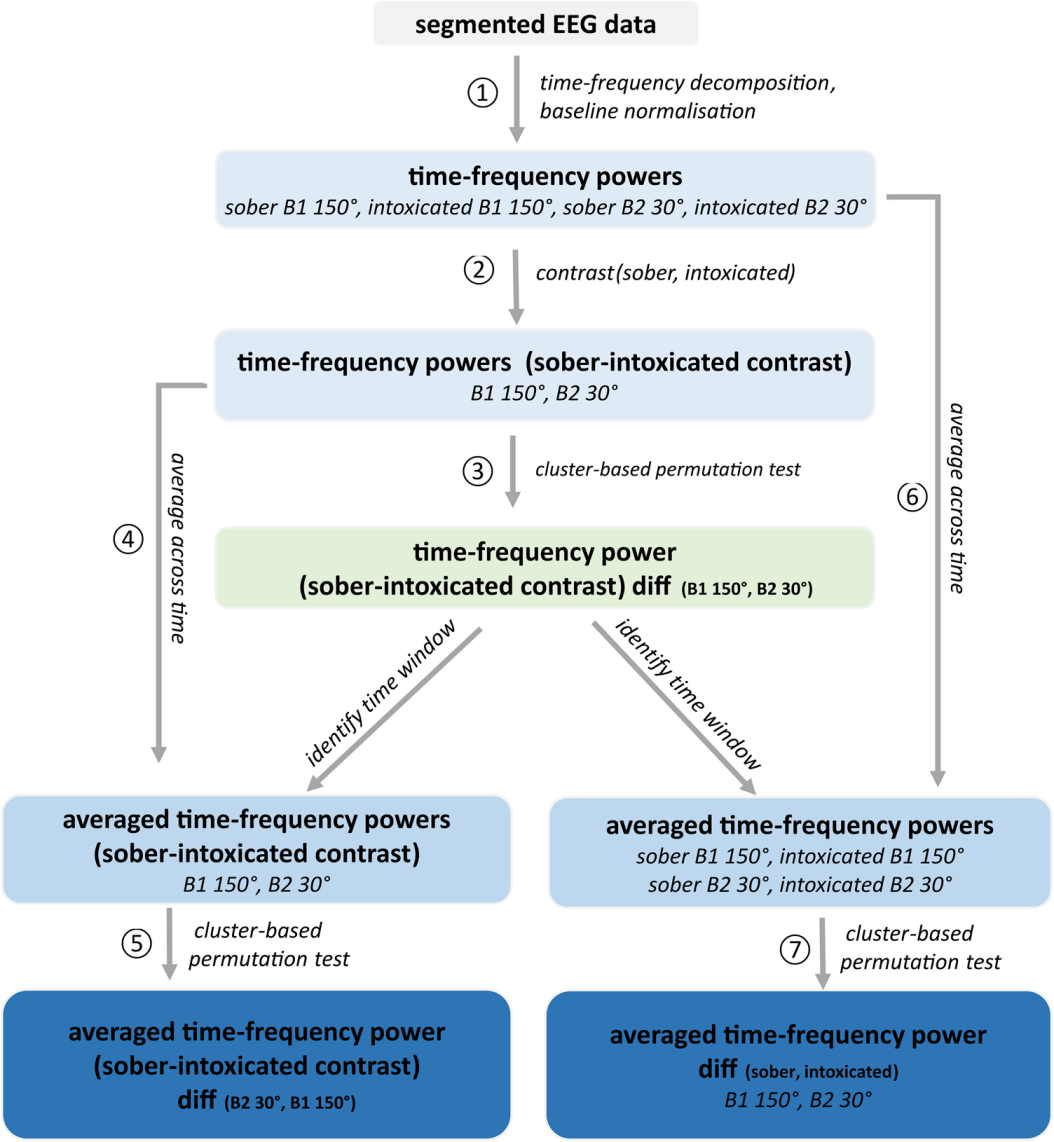
The illustration of procedures in time–frequency decomposition and cluster-based permutation tests for both theta and alpha band activities. Each box indicates one category of data with different colors denoting different data types. Each arrow indicates one operation/data transformation. The numbers indicate the order of steps. Please note that for the other two task conditions, basic data is provided in the Supplementary Files.

### Experimental design and procedures

The experimental paradigm was conducted twice, i.e., first in a sober and second in an intoxicated state. In this context, it needs to be noted that the initial study design comprised one pre-intoxication and one post-intoxication sober assessment. As those did not significantly differ from each other, we counter-balanced the appointment data in our initial study (so that the sober data of one half of the sample was taken from the pre-intoxication sober appointment and the other half was taken from the post-intoxication sober appointment). Yet, the post-intoxication appointment was usually conducted on the day following experimental intoxication and even though there had not been any significant behavioral differences between the sober assessments before vs. after experimental intoxication, we could not safely exclude the possibility that the neurophysiological data might indeed have slightly differed on the day following intoxication (despite the fact that the participants did typically not report to feel hungover on that day). For this reason, we decided to only use the T1/pre-intoxication data for the assessment of sobriety in our current study. Participants were asked to abstain from alcohol on the night before the sober assessment. On both appointments, the participants were required to not consume stimulant substances like caffeine, nicotine, guanine, etc. within 4 h before the experiment. No eating was allowed within 3 h before the intoxication appointment. To experimentally induce a binge-like alcohol intoxication of approximately 1.2‰ (120 mg/dl), an individual amount of vodka (40 Vol%) was calculated based on the estimated total body water and an assumed resorption deficit of 20%. The data sheet used to calculate the individual alcohol amount on site is provided as a Supplement. The vodka was mixed with orange juice in a ratio of 1:1 and served at room temperature. All participants were required to consume their drink within 30 min and wait for another 30 min after the end of consumption. Three episodes of Big Bang Theory were used to entertain the participants during the consumption and waiting period to avoid potential mood-swings and prevent the participants from engaging too much with the experimenters. BrAC was measured immediately before and after task performance using the “Alcotest 3000” analyzer (Drägerwerk, Lübeck, Germany).

The current study employed a combined Go/Nogo-mental rotation paradigm as applied in previous studies^16,28^. The task required the participants to either respond or restrain their motor response according to the features of the target stimuli. Each trial started with a fixation cross, which was presented for 800 ms. This was followed by a target stimulus presentation until a button was pressed (or 1100 ms had elapsed). Two different single digit numbers and single letters (5, 7, G, R) were used as target stimuli. Those specific stimuli had been chosen because they cause no gender differences in mental rotation^59^. The target was always rotated and either displayed in a mirrored or non-mirrored form. The rotation angle of each stimulus was 30°, 90°, or 150°, with larger rotation angles imposing higher working memory load^16^. In this context, it should be noted that differences in working memory load are considered to be a modulating factor, but not the dependent variable in this task. Non-mirrored stimuli were rotated clockwise and mirrored stimuli were rotated counter-clockwise.

The experiment consisted of two blocks with different task complexity. In the cognitively more challenging block 1, all trials with a non-mirrored stimulus indicated a Go trial (left button press for non-mirrored letters and right button press for non-mirrored numbers), and trials with mirrored stimuli were Nogo trials (no button press for mirrored numbers and letters). In block 2, a letter indicated a Go trial (left button press for mirrored letters and right button press for non-mirrored letters), and a number indicated a Nogo trial (no button press for mirrored and non-mirrored numbers). The ratio between Go and Nogo trials was 7:3 to increase the response tendency in Nogo trials^28^. The combinations of stimuli and corresponding responses in both blocks are illustrated in Fig. 4.

The two blocks were run consecutively on each appointment, with block 1 always preceding block 2. 360 trials (252 Go trials and 108 Nogo trials), were presented randomly in each block. The entire experimental paradigm consisted of 720 trials and lasted for about 30 min.

Of note, the standardized instruction and a 60-trial exercise to familiarize the participants with each block was always conducted while the participants were (still) sober and repeated right before task performance, whenever necessary.

### EEG recording and processing

EEG data were recorded from 60 equidistant Ag/AgCl electrodes using the BrainVision Recorder software package (BrainVision Recorder, Version 1.20.0601, Brain Products GmbH, Gilching, Germany). The coordinates of the ground and reference electrodes were theta = 58, phi = 78 and theta = 90, phi = 90, respectively. After recording and offline downsampling to 256 Hz, Infinite Impulse Response (IIR) filters from 0.5 to 20 Hz at a 48 dB/oct slope, and an additional notch filter of 50 Hz were applied on the EEG data. After that, defective channels were removed. Subsequently, a manual inspection and an Independent Component Analysis (ICA, infomax algorithm) were applied on the remaining channels to remove rare technical artifacts and regular artifacts like eye blinks and pulse artifacts. Afterwards, discarded channels were interpolated using neighboring electrodes, and a new average reference was calculated. After EEG pre-processing, the continuous EEG data were segmented into single trials. Each trial was locked to the stimulus onset with a length of 5000 ms, starting from 2000 ms before stimulus onset and ending 3000 ms after stimulus onset. Within these segments, trials with artifacts were rejected applying the following criteria: voltage difference in an interval of 200 ms higher than 150 µV; voltage step higher than 50 µV/ms; amplitude higher than 100 µV or lower than − 100 µV. Baseline correction was applied in the remaining trials using baseline activities between − 200 and 0 ms (i.e., directly before stimulus onset). Then, all baseline-corrected trials with correct responses were categorized according to the experimental factors: intoxication (sober vs. intoxicated), block (1 vs. 2), rotation angle (30°, 90°, and 150°), and condition (Go vs. Nogo). The further analyses excluded all trials with a 90° rotation angle because these stimuli may not require mental rotation, as humans are usually very familiar with such objects^28,60,61^. Interested readers can however find behavioral data on this condition in the Supplementary Materials. Also, all Go trials were excluded since this study focuses on response inhibition.

### Time–frequency decomposition

To examine how working memory load modulates the differences in intoxication effects on response inhibition, electrode-based analyses of the EEG data were only conducted for the cognitively easiest (B2 30°) and hardest (B1 150°) Nogo trials. This procedure maximizes effect variance and allows a reliable analysis. The data analysis steps are illustrated in Fig. 5. All methodological details concerning the single steps can be found in the Supplementary Material.

### Source estimation

A Dynamical Imaging of Coherent Sources (DICS) beamformer was used to identify the neuroanatomical source of the intoxication effect difference in response inhibition (Nogo trials) between the cognitively easiest (B2 30°) and hardest (B1 150°) conditions. Beamforming analyses were conducted separately for the theta band (4–7 Hz) and the alpha band (8–12 Hz). First, the frequency power and spectral density matrix for every single condition (sober B2 30°, intoxicated B2 30°, sober B1 150°, and intoxicated B1 150°) was calculated for each individual using a single taper (“Hanning”) for the baseline and post-stimulus activities, separately. The time window for the post-stimulus activity was set from 750 to 1500 ms after stimulus onset, as informed by the electrode-based analysis. The baseline activity was selected from − 750 to 0 ms relative to stimulus onset. Then, a common spatial filter was calculated using all estimated frequency representations and subsequently applied to each frequency band activity to extract the source. After that, the source powers of all conditions were baseline-normalized using a decibel transformation (see above). The source of intoxication effect differences between the most and least demanding conditions was calculated as (P_sober B1 150°_ − P_intoxicated B1 150°_) − (P_sober B2 30°_ − P_intoxicated B2 30°_) where P is power, and P_sober B1 150°_ − P_intoxicated B1 150°_/P_sober B2 30°_ − P_intoxicated B2 30°_ indicated intoxication effects in B1 150° and B2 30° conditions, respectively. Then, the source of the intoxication effect difference was estimated using the ‘standard_mri’ head model in FieldTrip for each participant. The grand-averaged difference was calculated by averaging the source power across all participants. We then performed cluster-based permutation tests at the electrode level. We chose the voxels showing powers higher than 70% of the maximum existent value to reconstruct the anatomical source of workload modulation on the intoxication effect.

### Statistical analyses

The statistical analysis of the behavioral data focused on the false alarm rate as an index of response inhibition performance. For each participant, the false alarm rate of every Nogo condition was calculated. A repeated measures ANOVA was applied using the within-subject factors “intoxication” (sober vs. intoxicated), “block” (1 vs. 2), and “rotation angle” (30° vs. 150°). A Greenhouse–Geisser correction was applied whenever necessary. Due to the non-normal distributions of most variables, as indicated by Komolgorov–Smirnov tests, additional non-parametric tests were applied to confirm significant effects obtained with parametric statistis.

### Software

BrainVision Recorder (Version 1.20.0601) [Software] (2013). Gilching, Germany: Brain Products GmbH. Manual and product information available at: http://sites.bu.edu/reinhartlab/files/2017/06/BrainVision_Recorder_UM-1.pdf.

## Supplementary Information

Supplementary Information 1.

Supplementary Information 2.

## Data Availability

The behavioral raw data and analyses as well as the scripts used for EEG analyses are available on the open science framework (https://osf.io/wbafc/?view_only=e98d0f3c75e14c8992ec24c4e3bb8b7f). The raw EEG data and code will be made available upon request.
