# Supplementary material for: How low working memory demands and reduced anticipatory attentional gating contribute to impaired inhibition during acute alcohol intoxication
Source: Sci Rep. 2022 Feb 21;12:2892. doi: 10.1038/s41598-022-06517-9 (PMC8861183; doi:10.1038/s41598-022-06517-9)
Supplement: Supplementary file 2 — Supplementary Information 2. [file 41598_2022_6517_MOESM2_ESM.pdf]

Supplementary Material

Time-Frequency Decomposition: Details

In the first step, time-frequency power spectra of single trials were calculated using Morlet wavelets and separately averaged across trials for each condition and participant. Then, averaged powers were normalized using decibel transformation (dB Power = $10 \times \log_{10}(\text{Power}_{\text{activity}}/\text{Power}_{\text{baseline}})$) to obtained information about event-related synchronization and desynchronization processes in the analyzed frequency bands. Baseline power was calculated as the average power in the time window from 200 to 0ms before stimulus onset. Then, theta (4 - 7Hz) and alpha (8 - 12 Hz) band powers were averaged across the respective frequency bands for each condition (sober B1 150°, intoxicated B1 150°, sober B2 30°, and intoxicated B2 30°) (step 1). Further analyses were conducted for theta and alpha band powers separately. To examine the intoxication effect, we computed the time-frequency power difference between the sober and the intoxicated conditions (step 2). This was done separately for the least demanding condition (B2 30°) and the most demanding condition (B1 150°). For each frequency band, the time-frequency representations of the intoxication effect were then compared between the B2 30° and B1 150° condition using cluster-based permutation tests (step 3). Hence, we identified the time points where significant intoxication-related differences between the B2 30° and the B1 150° condition occurred in the theta and alpha bands. The further analyses employed time-frequency representations of these sober-intoxicated contrasts (B2 30°, B1 150°) and the original conditions (sober B1 150°, intoxicated B1 150°, sober B2 30°, and intoxicated B2 30°) within the selected time window and separate cluster-based permutation tests were applied to identify electrodes showing significant effects. Using the sober-intoxicated contrasts, we aimed to examine how different working memory load modulates the intoxication effect. Here, we selected the time-frequency representations of sober-intoxicated contrasts in the time window in which most electrodes revealed a significant B2 30°-B1 150° difference in the theta and alpha band and averaged them across time (step 4). Then, a cluster-based permutation test was applied to identify the electrodes showing a significant difference between conditions B2 30° and B1 150° in the selected time window (step 5). Using the time-frequency representations of the original four conditions, we aimed to examine where the difference of intoxication effects between the B2 30° and B1 150° was evident at the electrode level. To do that, we averaged the original time-frequency representations across the same selected window (step 6) for each condition and compared the averaged time-frequency representations in two pairs (sober B1 150° and intoxicated B1 150°; sober B2 30° and intoxicated B2 30°) using a cluster-based permutation test (step 7).

All cluster-based permutation tests were based on dependent t-tests for each electrode (and time points in step 3). We used the Monte-Carlo method to compute the reference distribution of the permutation test with 1000 random draws. The cluster-level t values were computed using the sum of all t-values within electrodes (and time points in step 3).

Behavioral results of the 90° condition

In order to provide a full picture, we ran an add-on repeated measures ANOVA for the false alarm rates of the 90° rotation condition, with “intoxication” (sober vs. intoxicated) and “block” (block 1 vs. block 2) as within-subject factors. This yielded a significant main effect of "block" ($F(1,19)=13.83$, $p=.001$, $\eta_p^2=.42$), with higher false alarm rates in the most demanding block 1 (9.58%±2.12) than in the least demanding block 2 (1.94%±0.74). Since none of those false alarm rates were normally distributed, we confirmed this difference with a Wilcoxon test for paired samples ($p<.001$).The behavioral raw data and analyses are available on the open science framework (https://osf.io/wbafc/?view_only=e98d0f3c75e14c8992ec24c4e3bb8b7f).

Behavioral side and switching effects

From block 1 to block 2, half of the stimuli switched response category (from Go to Nogo or vice versa) and the other half of the Go stimuli required a different side response (specifically, non-mirrored letter stimuli required a left hand Go response in block 1 and a right hand Go response in block 2) (compare Fig. 4 in the main text). In order to investigate whether this switch in the required Go response led to different performance, we ran an exploratory repeated measures ANOVA on Go accuracy measures (averaged over the 30° and 150° conditions) using the factors "intoxication" (sober vs. intoxicated), "block" (1vs. 2), and "correct response side" (left vs. hand). Doing so, we found main effects of intoxication (F(1,19)=12.41, p=.002, η_p^2=.40, sober=90.91%±1.27, intoxicated=85.80%±2.08), block (F(1,19)=4.56, p=.046, η_p^2=.19, block 1=89.97%±1.64, block 2=86.74%±1.83), and correct response side (F(1,19)=6.80, p=.017, η_p^2=.26, left=89.60%±1.57, right=87.11%±1.69). As not all of the variables were normally distributed, all of those differences were confirmed by Wilcoxon tests (all p ≤.037). However, we did not find any significant interaction effects (all F(1,19)≤4.33, all p≥.051), which means that we did not find significant evidence for the assumption that switching response sides between block 1 and block 2 impaired correct responding, or was differentially modulated by intoxication.

We further investigated potential differences in Nogo performance in block 2, as half of the Nogo stimuli had already been used as Nogo stimuli in block 1, while the other half had been used as Go stimuli in block 1 (compare Fig. 4 in the main text). We ran an exploratory repeated measures ANOVA on block 2 Nogo false alarm rates (averaged over the 30° and 150° conditions) using the within-subject factors "intoxication" (sober vs. intoxicated) and “Nogo stimulus” (repeated vs. switched in relation to block1). Doing so, we found a main effect of intoxication (F(1,19)=7.39, p=.014, η_p^2=.28, sober=0.14%±0.10, intoxicated=2.08%±0.71), which was confirmed by a Wilcoxon test (p=.003). However, we found no main or interaction effect of Nogo stimulus (all F(1,19)≤0.26, all p≥.618), which means that we did not find significant evidence for the assumption that switching Go and Nogo stimuli between block 1 and block 2 impaired correct responding in Nogo trials, or was differentially modulated by intoxication.

Neurophysiology of the two other task conditions (B1 30° and B2 150°)

Task-related/baseline-corrected theta and alpha band powers as well as the intoxication effects in the task conditions not investigated / discussed in the main manuscript are provided in Figure S1.

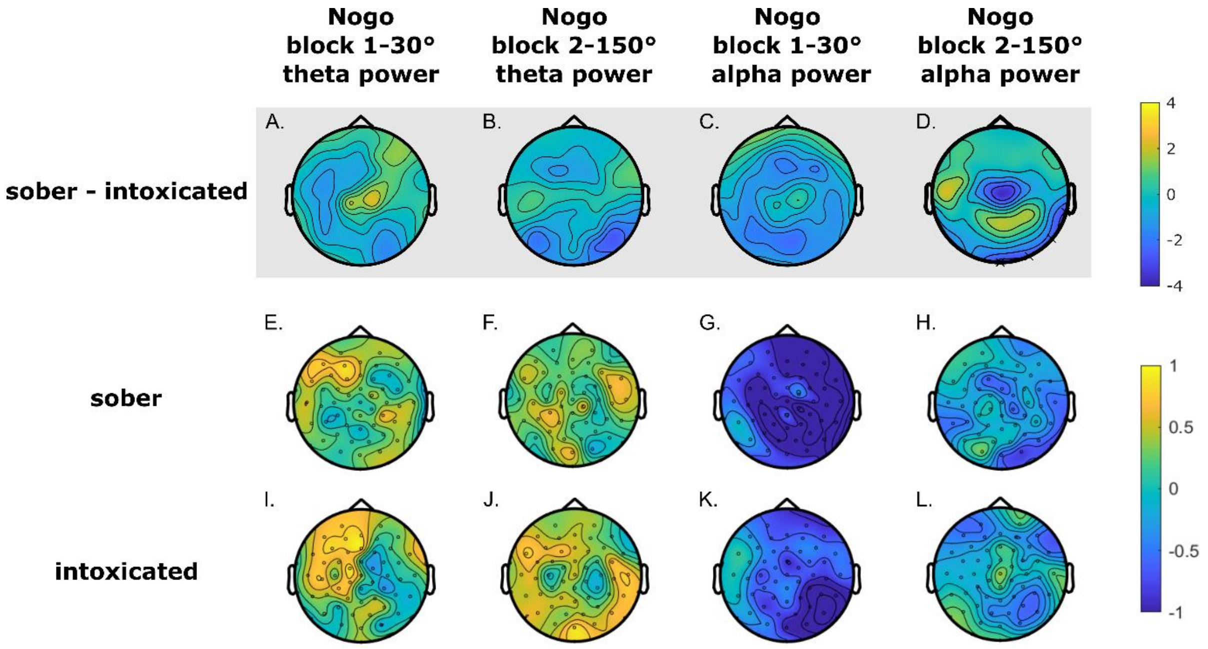

Figure S-1. Neurophysiological results of task-related theta and alpha activity of Nogo trials. The top row presents the topographical maps of intoxication effects in the conditions of block 1 at 30° and block 2 at 150° separately for the alpha and theta bands. Warm and cold colors indicate the positive and negative contrasts of task-related alpha and theta band powers (sober-intoxicated). The color bar indicates the t-values of cluster-based permutation tests and '×' marks the electrodes with significant differences of $p \leq .05$. The middle and lower rows show the topographical maps of task-related alpha and theta activities in the original conditions. The color bar indicates baseline-normalized power in dB. All presented results were derived from data between 0.75 to 1.5s after stimulus presentation.
